# Evolution of the key alkaloid enzyme putrescine *N*-methyltransferase from spermidine synthase

**DOI:** 10.3389/fpls.2013.00260

**Published:** 2013-07-29

**Authors:** Anne Junker, Juliane Fischer, Yvonne Sichhart, Wolfgang Brandt, Birgit Dräger

**Affiliations:** ^1^Faculty of Science I, Institute of Pharmacy, Martin-Luther-University Halle-WittenbergHalle (Saale), Germany; ^2^Department of Bioorganic Chemistry, Leibniz Institute of Plant BiochemistryHalle (Saale), Germany

**Keywords:** spermidine synthase, putrescine *N*-methyltransferase, evolution, *Datura stramonium*, *Arabidopsis thaliana*, homology modeling

## Abstract

Putrescine *N*-methyltransferases (PMTs) are the first specific enzymes of the biosynthesis of nicotine and tropane alkaloids. PMTs transfer a methyl group onto the diamine putrescine from *S*-adenosyl-l-methionine (SAM) as coenzyme. PMT proteins have presumably evolved from spermidine synthases (SPDSs), which are ubiquitous enzymes of polyamine metabolism. SPDSs use decarboxylated SAM as coenzyme to transfer an aminopropyl group onto putrescine. In an attempt to identify possible and necessary steps in the evolution of PMT from SPDS, homology based modeling of *Datura stramonium* SPDS1 and PMT was employed to gain deeper insight in the preferred binding positions and conformations of the substrate and the alternative coenzymes. Based on predictions of amino acids responsible for the change of enzyme specificities, sites of mutagenesis were derived. PMT activity was generated in *D. stramonium* SPDS1 after few amino acid exchanges. Concordantly, *Arabidopsis thaliana* SPDS1 was mutated and yielded enzymes with both, PMT and SPDS activities. Kinetic parameters were measured for enzymatic characterization. The switch from aminopropyl to methyl transfer depends on conformational changes of the methionine part of the coenzyme in the binding cavity of the enzyme. The rapid generation of PMT activity in SPDS proteins and the wide-spread occurrence of putative products of *N*-methylputrescine suggest that PMT activity is present frequently in the plant kingdom.

## Introduction

Plants contain an unparalleled variety of chemical structures, and many of the compounds are utilized as aromas, dyes and medicines. The diverse chemicals are synthesized mostly in individual plant genera by several specialized enzymatic steps. The evolution of the biosynthetic machineries, the enzymes in particular that produce those many compounds, is largely not understood.

**Figure 1:**
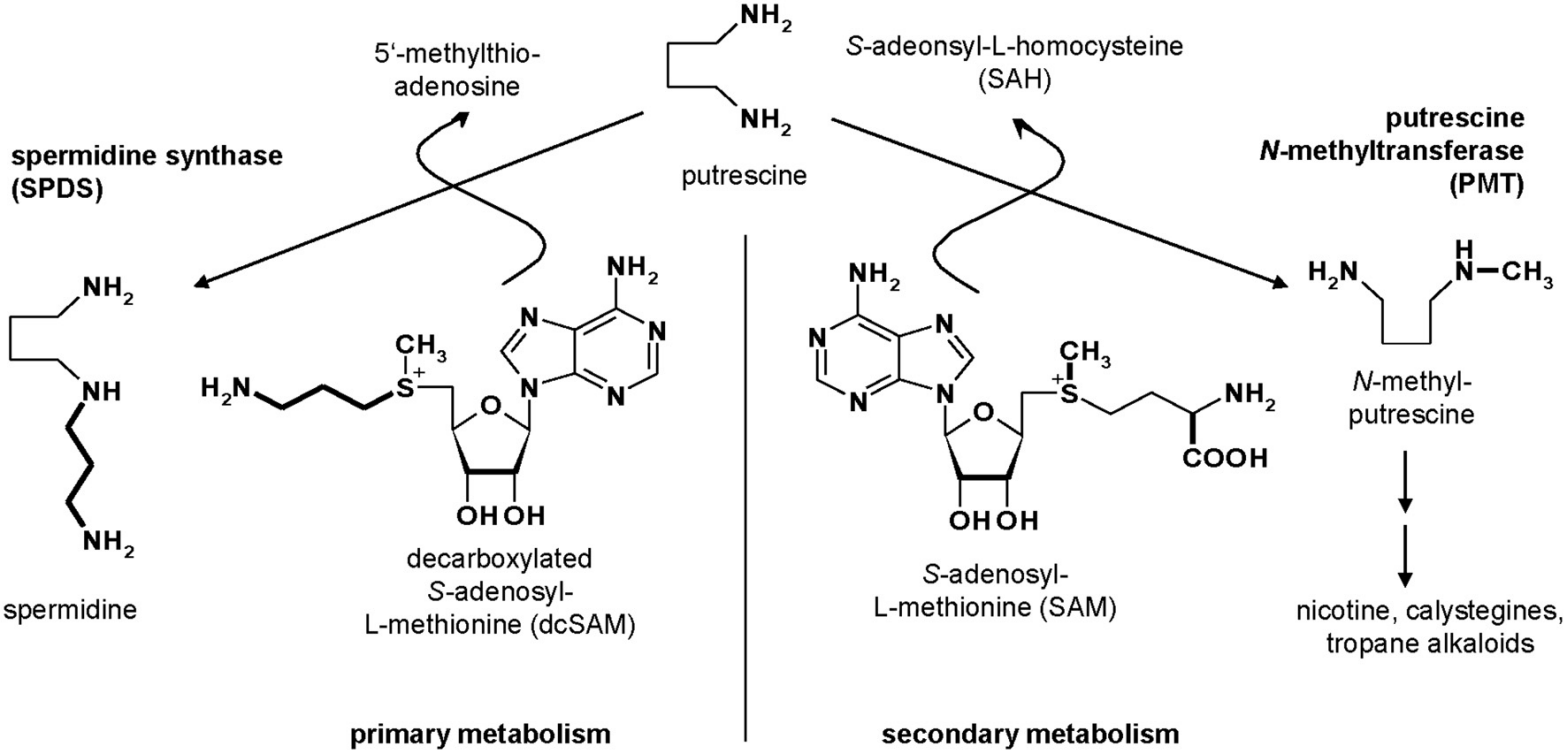
**Metabolism of putrescine**.

Putrescine *N*-methyltransferase (PMT, E.C. 2.1.1.53) is the first specific enzyme of tropane, nortropane, and nicotine biosynthesis in Solanaceae and Convolvulaceae (Biastoff et al., 2009a). Alkaloids of this large group are essential sympatholytic drugs used clinically like atropine and scopolamine. These alkaloids also confer advantages to the plants that produce them (Berenbaum, 1995). Cocaine is an example for a tropane alkaloid acting as strong insecticide (Nathanson et al., 1993). Nicotine deters all kinds of herbivores (Baldwin, 1998), and hyoscyamine reduces attack of generalist herbivorous insects (Shonle and Bergelson, 2000). PMT as key enzyme for those alkaloids transfers a methyl group from *S*-adenosyl-l-methionine (SAM) to the ubiquitous diamine putrescine, forming *N*-methylputrescine (Figure 1). PMTs show higher amino acid sequence similarity to spermidine synthases (SPDSs, E.C. 2.5.1.16) than to other plant methyltransferases (Stenzel et al., 2006). SPDSs are ubiquitous enzymes of primary metabolism and use the same substrate putrescine for an aminopropyl transfer from decarboxylated SAM (dcSAM) forming spermidine, an essential polyamine in all eukaryotic organisms.

The overall similarity between SPDSs and PMTs indicates the evolutionary origin of specialized PMT from ubiquitous SPDS by gene duplication and a change of function (Hashimoto et al., 1998b; Minguet et al., 2008). The change of a gene function appears a rare event in nature, because a gene duplicate most frequently will accumulate mutations and become a pseudogene, which is rapidly lost from the genome (Zou et al., 2009). The concept of gene duplication and change of function therefore necessitates a rapid shift from the initial function to the new function that promotes the genetic fixation of the new gene and avoids the loss of the gene, a demand that appears in conflict with the randomness of mutations. Even if a duplicated gene encoding an enzyme remains to be transcribed and translated, enzymes are sophisticated catalysts in which most mutations will lead to a loss of enzymatic activity or even to a loss of protein structure inducing degradation (Tokuriki and Tawfik, 2009). Multifunctional enzymes are postulated as evolutionary ancestors for enzymes of specialized metabolism (Aharoni et al., 2005). All extant SPDSs, however, are specific for decarboxylated SAM and do not transfer a methyl moiety (Ikeguchi et al., 2006).

For SPDS proteins, several protein structures with ligands obtained from crystal X-ray and available in the protein database (Berman et al., 2000), e.g., from *Thermotoga maritima* (Korolev et al., 2002), *Plasmodium falciparum* (Dufe et al., 2007), *Homo sapiens* (Wu et al., 2007), and *Escherichia coli* (Zhou et al., 2010) enabled to locate binding regions for substrate and coenzyme. Active site definition of SPDS focused on 20 amino acids participating in substrate and coenzyme binding (Korolev et al., 2002). A few amino acids in the active site differ systematically between SPDSs and PMTs. X-ray structure elucidation for PMT failed up to now, but ca. 60% overall sequence identity between PMTs and plant SPDSs enabled homology modeling of *Datura stramonium* PMT (DsPMT). A similar protein fold, the Rossman fold, consisting of seven β-sheets and six α-helices (Kozbial and Mushegian, 2005) was obtained for PMT. Two chimeric proteins composed of *D. stramonium* SPDS1 and DsPMT N-terminal and carboxyl terminal parts and *vice versa* were active as PMT and SPDS, respectively, and localized the amino acids decisive for specific catalytic activity in the N-terminal parts of both proteins (Biastoff et al., 2009b). Alterations of the coenzyme binding region were suggested to change the selectivity for SAM vs. dcSAM (Ikeguchi et al., 2006; Minguet et al., 2008). The feasibility for such an activity transit in the SPDS protein fold was never shown experimentally.

With an interest to understand how the enormous metabolic diversity in plants may have evolved, we attempted to identify possible and necessary single steps in the evolution of PMT from SPDS. Formally, this process requires SPDS to switch the coenzyme preference from dcSAM to SAM and to continue accommodating putrescine. SAM binding not only necessitates an enlarged binding site for the additional carboxyl group of SAM. The chiral sulfur in dcSAM and SAM determines the orientations of the aminopropyl group and of the methyl group. For a successful transit in catalysis, the methyl group to be transferred by PMT instead of the aminopropyl group transferred by SPDS must be moved into sufficient proximity to the putrescine nitrogen. Here, we took advantage of protein homology models and ligand docking to identify amino acids for site directed mutagenesis. We switched the activity of SPDS from aminopropyl transfer to methyl transfer by exchange of a few amino acids. The transit was equally successful for SPDS from a tropane alkaloid producing Solanaceae, *D. stramonium*, and for SPDS from *Arabidopsis thaliana*, a non-related plant that does not contain tropane or nicotine alkaloids (Brock et al., 2006; Ikeguchi et al., 2006).

## Materials and methods

### Site directed mutagenesis

Wild type cDNA of *spds1* (Accession No: Y08252) and *pmt* (Accession No. AJ583514) of *D. stramonium* were cloned into pET-21 d vector (Novagen) and synthesized in *E. coli* Bl21-CodonPlus (DE3)-RP (Stratagene) with a hexa-histidine tag (his-tag) at the C-terminus. Wild-type cDNA of *A. thaliana spds1* (Accession No: AJ251296) was cloned into pQE-30 (QIAGEN) and synthesized in M15[pREP4] cells (QIAGEN) with an N-terminal his-tag. Mutagenesis PCR was done according to the QuikChange II Site Directed Mutagenesis Kit (Stratagene). All intended mutations were confirmed by sequencing. Primers are listed in Table 1.

**Table 1:** **Primers for mutagenesis of DsPMT, DsSPDS1 and AtSPDS1, mutated triplets are underlined**.

**Exchanged amino acids**	**Mutagenesis primers 5′–3′**	
**DsPMT**		
H108L	Fwd	GGATGGAGCAATTCAACTCACAGAGAATGGTGGATTTC
	Rev	GAAATCCACCATTCTCTGTGAGTTGAATTGCTCCATCC
**DsSPDS1**		
L70H	Fwd	GGTGTGATACAACACACAGAGCGGGATG
	Rev	CATCCCGCTCTGTGTGTTGTATCACACC
Q79T	Fwd	GATGAATGTGCTTACACAGAAATGATCACTCATCTCC
	Rev	AGATGAGTGATCATTTCTGTGTAAGCACATTCATCCC
D103I	Fwd	GTTATTGGAGGAGGTATTGGTGGTGTCTTGCGTG
	Rev	CACGCAAGACACCACCAATACCTCCTCCAATAAC
V106T (with D103I)	Fwd	GGAGGTATTGGTGGTACCTTGCGTGAGGTGTCTCG
	Rev	CGAGACACCTCACGCAAGGTACCACCAATACCTCC
**AtSPDS1**		
L98H	Fwd	GTTTTGGATGGAGTAATCCAACATACGGAGAGAGATG
	Rev	CATCTCTCTCCGTATGTTGGATTACTCCATCCAAAAC
Q107T	Fwd	GAGAGAGATGAATGTGCTTATACGGAAATGATCACTCATCTTCC
	Rev	GGAAGATGAGTGATCATTTCCGTATAAGCACATTCATCTCTCTC
D131I	Fwd	GTCATTGGAGGAGGAATTGGAGGTGTCCTGCGG
	Rev	CCGCAGGACACCTCCAATTCCTCCTCCAATGAC
V134T (with D131I)	Fwd	GGAGGAATTGGAGGTACCCTGCGGGAAGTTGC
	Rev	GCAACTTCCCGCAGGGTACCTCCAATTCCTCC
D204A	Fwd	GTTATTGTTGACTCTTCAGCTCCAATCGGTCCTG
	Rev	CAGGACCGATTGGAGCTGAAGAGTCAACAATAAC
D204K	Fwd	GATGCAGTTATTGTTGACTCTTCAAAACCAATCGGTCCTGC
	Rev	GCAGGACCGATTGGTTTTGAAGAGTCAACAATAACTGCATC
E236A	Fwd	GTGTGCACTCAAGCTGCGAGCTTGTGGCTTCAC
	Rev	GTGAAGCCACAAGCTCGCAGCTTGAGTGCACAC
Y270A	Fwd	CAGCGTTCCAACAGCCCCCAGTGGGGTCAT
	Rev	ATGACCCCACTGGGGGCTGTTGGAACGCTG

### Protein synthesis and purification

Protein was synthesized at 37°C for 4–6 h after induction with 1 mM isopropyl β-D-1-thiogalactopyranoside. Bacteria were lysed in buffer A (50 mM sodium phosphate, 300 mM sodium chloride, 10 mM imidazole, pH 8.0) with Sigma protease inhibitor cocktail for proteins with his-tags and 800 μg ml^−1^ lysozyme by sonication (Branson Sonifier 250). After addition of 10 μg ml^−1^ DNase I, centrifugation (40 min, 15000 g, 4°C), and filtration (0.45 μm, cellulose acetate), the supernatant was applied on a HisTrap HP 1 ml column (GE Healthcare), equilibrated with buffer A. Protein was eluted using a gradient of 10–500 mM imidazole. The imidazole buffer was directly exchanged by storage buffer B (20 mM HEPES, pH 8.0, 2 mM dithiothreitol, 1 mM ascorbic acid) on PD-10 desalting columns (Sephadex G-25, GE Healthcare). Purified proteins were stored at −80°C after addition of 10% (per volume) glycerol. Protein concentrations were determined with Coomassie Brilliant Blue with bovine serum albumin as standard (Bradford, 1976).

### Enzyme activity and kinetic measurement

Standard enzyme assays contained up to 200 μg purified protein in 20 mM HEPES, pH 8.0 with 2 mM dithiothreitol and 1 mM ascorbic acid in a total volume of 250 μl. 0.01–10 mM putrescine as substrate and 0.01–1 mM SAM or 1 mM dcSAM as coenzyme were added. Protein amounts for kinetic measurements were adapted according to protein and reaction time linearity. Enzyme assays were incubated for 30 min at 37°C and stopped by alkalinization. Polyamines must be free bases to undergo complete derivatization with dansyl chloride, but concentrated sodium hydroxide used before in the solution stopping the enzyme reaction (Teuber et al., 2007) interferes with the sulfonamide formation. Saturated sodium carbonate solution was used instead (Smith and Davies, 1985; Marce et al., 1995). Glycine in the assay buffer was exchanged for HEPES buffer, which did not influence the derivatization reaction. Two hundred to four hundred microliters of the alkaline mixture were dansylated as described (Smith and Davies, 1985). Dansylated *N*-methylputrescine and spermidine were separated by HPLC and quantified using fluorimetric detection (365 nm excitation, 510 nm emission) in addition to diode array detection (Marce et al., 1995). Detection limits for assays containing 200 μg protein and using 400 μl alkaline mixture for dansylation were 1.36 pkat mg^−1^ for PMT and 0.96 pkat mg^−1^ for SPDS. Each assay was repeated four times. Kinetic parameters were computed by SigmaPlot 10.0, enzyme module 1.3 (Systat Software).

### Molecular modeling

Due to the slightly differently oriented gate keeping loop in the X-ray structure of AtSPDS1 (PDB: 2Q41) (Levin et al., 2007) in comparison to the human SPDS (Wu et al., 2007), a homology model of AtSPDS1 was built and used for further analysis. The AtSPDS1 as well as DsPMT homology models were created based on human SPDS (PDB: 2O0L chain B) using MOE (Molecular Operating Environment, 2011.10; Chemical Computing Group Inc., 2011) and subsequent molecular dynamics refinement by YASARA 11.4.18 2011 (Krieger et al., 2002) with the Yasara2 force field (Krieger et al., 2009). The positions of dcSAM and putrescine in AtSPDS1 model were congruent in SPDS PDB structures [human 2O0L containing dcSAM and 2O06 with co-crystallized putrescine (Wu et al., 2007), *P. falciparum* 2PT6 with dcSAM (Dufe et al., 2007)] and taken accordingly. SAM in DsPMT was manually constructed from dcSAM using the builder of MOE. The model of DsSPDS1 (Biastoff et al., 2009a) was also subjected to molecular dynamics refinements in YASARA. Subsequently, all models were energy minimized with the Yasara2 force field. The three models were evaluated with PROCHECK (Laskowski et al., 1993) and PROSA II (Sippl, 1990, 1993). All models showed more than 86% of the amino acids in the most favored regions of the Ramachandran plot and no outlier (Table 2). The combined z-scores (Table 2) of PROSA II indicated a native-like fold. To explain the results of mutations, molecular dynamics simulation with the Amber03 (Duan et al., 2003) force field of SPDS mutants and of DsPMT wild type up to a simulation time of 10 ns were performed using YASARA. The transition state search for calculation of the *S*-SAM to *R*-SAM inversion barrier was carried out with JAGUAR (Version 7.8, Schrödinger, LLC, New York, NY, 2011) with *ab inito* LMP2, basis set 6-311G**, using *S*-SAM and *R*-SAM as input coordinates. The final models were accepted and deposited at the Protein Model DataBase PMDB (http://mi.caspur.it/PMDB/main.php) (Castrignano et al., 2006) and received the PMDB codes: AtSPDS1 PM0078550, DsPMT PM0078552, DsSPDS1 PM0078555.

**Table 2:** Ramachandran plot analysis with PROCHECK^*^ and combined *z*-score resulting from PROSA II.

**Protein**	**Most favored**	**Additional allowed**	**Generously allowed**	**Combined *z*-score**
AtSPDS	91.9	7.7	0.4	−10.11
DsPMT	86.2	13.4	0.4	−9.33
DsSPDS1	89.7	9.5	0.8	−9.82

* Results of the Ramachandran plot with rates (given in %) of residues in most favored, additional allowed, generously allowed region. Glycine and proline residues are not contained in these rates.

### Phylogenetic tree

The unrooted phylogenetic tree was computed with MEGA5 Software (Tamura et al., 2011). The Neighbor-Joining (NJ) method was used. In the phylogenetic investigation, 27 SPDS sequences, 14 PMT sequences, and 5 *N*-methyltransferase (NMT) sequences were analyzed (Accession numbers: Table 3). Only full-length amino acid sequences were included and aligned with Clustal W2 (Gonnet matrix). In consequence of several gaps in the alignment, pairwise deletion was used. Bootstrap values (1000 replicates) were indicated next to the branch points of the phylogenetic tree. Poisson correction model for amino acid exchange proportions was applied in NJ trees.

**Table 3:** **Accession numbers of sequences used for the phylogenetic tree in alphabetical order**.

**Protein**	**Accession number**	**Protein**	**Accession number**
			
*Anisodus tanguticus* PMT	AAT99576	*Malus domestica* SPDS1	Q8GTQ6
*Arabidopsis thaliana* SPDS1	AJ251296	*Nicotiana attenuata* PMT1	Q93XQ5
*Atropa belladonna* PMT1	Q9S7W8	*Nicotiana benthamiana* PMT	ABY25273
*Bacillus subtilis* SPDS	P70998	*Nicotiana sylvestris* PMT1	Q9ZWT9
*Bradyrhizobium japonicum* NodS NMT	Q9AQ22	*Nicotiana sylvestris* SPDS	O48660
*Caenorhabditis elegans* SPDS	Q9U2F0	*Nicotiana tabacum* PMT1	Q42963
*Calystegia sepium* PMT	Q2KTH2	*Nicotiana tabacum* SPDS	AAQ14853
*Cochlearia danica* SPDS	to be submitted	*Olea europaea* SPDS	ACZ73829
*Cochlearia officinalis* SPDS	CAO02391	*Oryza sativa* SPDS1	Q9SMB1
*Coffea arabica* SPDS	O82147	*Panax ginseng* SPDS	ACT21542
*Coffea canephora* DXMT	A4GE70	*Physalis divaricata* PMT	Q2KTH0
*Coffea canephora* XMT	A4GE69	*Physcomitrella patens* (predicted protein)	XP_001752964
*Cucumis sativus* SPDS	AAT66041	*Pisum sativum* SPDS1	Q9ZTR1
*Datura stramonium* PMT	Q70EW6	*Plasmodium falciparum* SPDS	CAB71155
*Datura stramonium* SPDS1	Q96556	*Ricinus communis* SPDS (putative)	XP_002534321
*Escherichia coli* SPDS	AAA24643	*Saccharomyces cerevisiae* SPDS	Q12074
*Helicobacter pylori* SPDS	O25503	*Scopolia parviflora* PMT (putative)	Q4VQ73
*Homo sapiens* HNMT	P50135	*Solanum dulcamara* PMT	CAQ19733
*Homo sapiens* NNMT	P40261	*Solanum lycopersicum* PMT	Q2KTH4
*Homo sapiens* SPDS	P19623	*Solanum lycopersicum* SPDS	Q9ZS45
*Hyoscyamus niger* PMT	Q9XJ41	*Solanum tuberosum* PMT	Q70AR0
*Hyoscyamus niger* SPDS1	O48658	*Solanum tuberosum* SPDS	Q93X16
*Lotus japonicus* SPDS	CAM35497	*Thermotoga maritima* SPDS	Q9WZC2

HNMT, histamine N-methyltransferase; NNMT, nicotinamide N-methyltransferase; NodS NMT, Nodulation protein S (N-methyltransferase); DXMT, 3,7-dimethylxanthine N-methyltransferase; XMT, Xanthosine methyltransferase.

## Results

### Hypothesis of SAM binding

The group transfer, either methyl or aminopropyl, requires a short distance between the group to be transferred and the reacting amino group of putrescine and correct position of the lone pair of the nitrogen atom for a S_*N*_2 reaction. For methyl transfer, the chiral sulfur of SAM must take an orientation different from that in dcSAM, or the substrate putrescine must change places. This consideration motivated the earlier hypothesis of a binding location for putrescine in PMT different from SPDS (Biastoff et al., 2009b). Other scenarios to achieve close vicinity between the putrescine nitrogen and the methyl group of SAM staying in the place of dcSAM can be imagined. An inversion of the sulfur chirality from *S*-SAM to *R*-SAM and thereby an orientation of the methyl group toward putrescine should be examined. Alternatively, conformational changes of the methionine moiety of SAM residing in the dcSAM position should be evaluated. A different configuration at the sulfur or different conformation of SAM may enable methyl transfer to putrescine, which equally stays at the position that it takes in SPDS.

**Figure 2:**
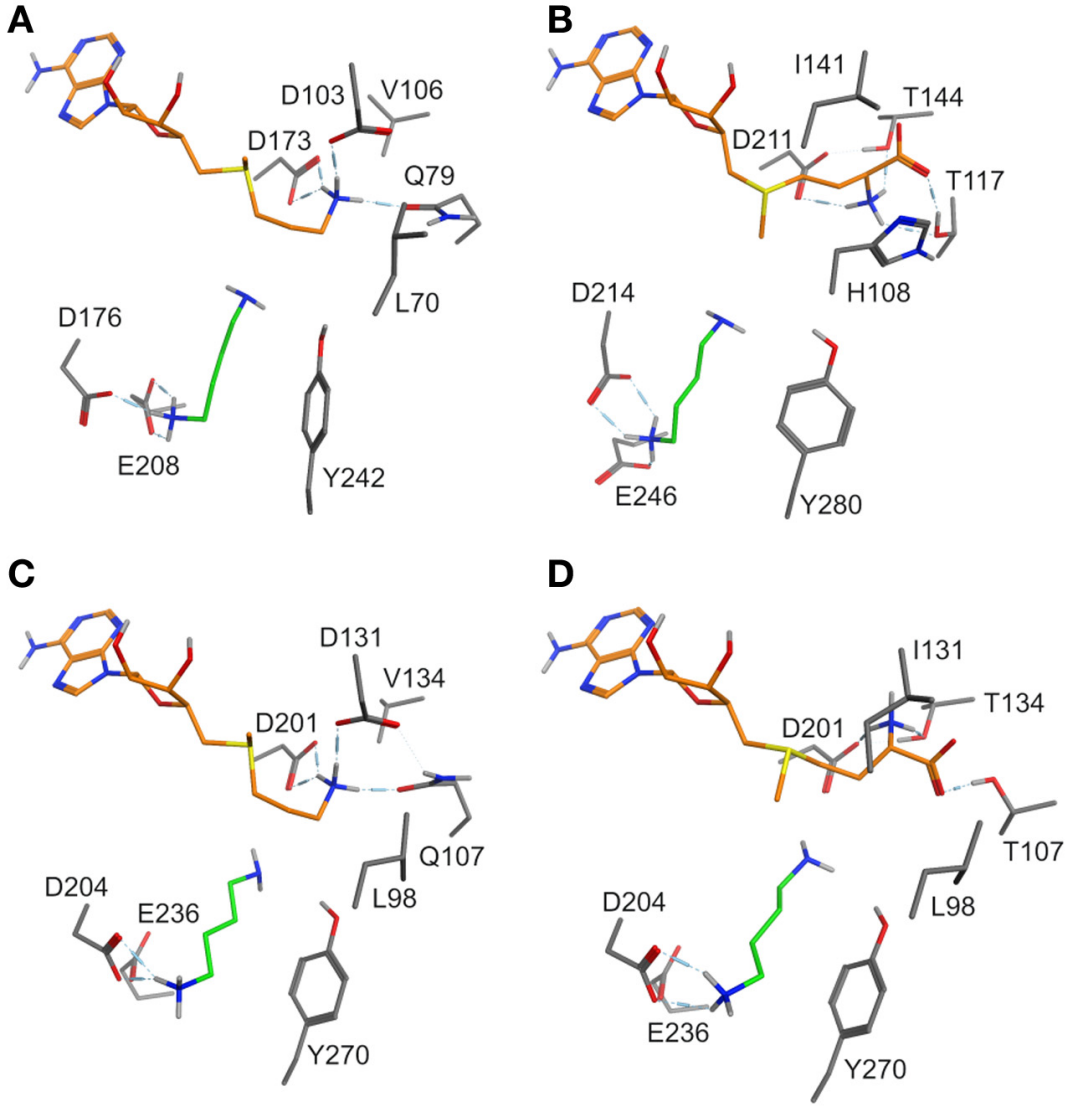
**(A)** Binding mode of putrescine (green carbon atoms) and of the methionine moiety (orange carbon atoms) of dcSAM in DsSPDS1 wild type, **(B)** SAM in DsPMT, **(C)** dcSAM in AtSPDS1 wild type, and **(D)** SAM in AtSPDS1 mutant with exchanges D131I, Q107T, V134T. For clarity, all non-polar hydrogen atoms are not displayed.

A switch of the sulfur chirality for methyl transfer instead of aminopropyl transfer would need a low inversion barrier. *Ab inito* calculations revealed a barrier of 30 kcal mol^−1^ and ruled out this option. For the evaluation of possible SAM conformations in enzymes, several coenzyme binding sites in *N*-methyltransferases co-crystallized with *S*-adenosyl-l-homocysteine were compared: nicotinamide *N*-methyltransferase (PDB: 3ROD) and histamine methyltransferase (PDB: 2AOT), both human, 1,7 dimethylxanthine methyltransferase (PDB 2EFJ) and xanthosine methyltransferase (PDB 2EG5), both from *Coffea*, and *N*-methyltransferase NodS from *Bradyrhizobium* (PDB: 3OFK) (Horton et al., 2005; McCarthy and McCarthy, 2007; Cakici et al., 2010; Peng et al., 2011). In these X-ray derived structures the positioning of the SAM adenosyl moiety is similar. The hydroxyl groups of ribose are stabilized by glutamate or aspartate with hydrogen bonds. Amino acids participating in binding of the methionine moiety are not conserved and vary between *N*-methyltransferases. Comparing the coenzyme binding regions of dcSAM and SAM in the SPDS and PMT models, amino acids recognizing the adenosyl moiety are highly conserved. Exchanges in the (decarboxy)methionine binding region are obvious, e.g., Q79T, D103I, V106T, and L70H (numbering following *D. stramonium* SPDS1), which may affect coenzyme fixation. The crystal structure-derived model of human SPDS (Wu et al., 2007) shows the amino group of the decarboxylated methionine forming salt bridges with amino acids corresponding to Q79, D103, and D173 and directing the C3 of the aminopropyl group in a position close to the amino group of the bound putrescine to allow aminopropyl transfer (shown for the DsSPDS1 model in Figure 2A).

Models of DsSPDS1 and DsPMT (Figures 2A,B) offered recommendations for mutagenesis to achieve methyl transfer activity. Due to the highly conserved residues in the adenosyl binding site SAM should bind similarly to dcSAM in SPDS. The conformational fixation of the aminopropyl moiety of dcSAM should be reduced by the substitution D103I (numbering following DsSPDS1) corresponding to the conserved isoleucine in DsPMT. D103 was also assumed to repel the carboxyl group of SAM (Korolev et al., 2002; Ikeguchi et al., 2006). Isoleucine instead of aspartate will exert a steric and electronic repulsion of the amino group necessitating a rotation of this group. The additional substitution Q79T will support a conformational change of methionine, if placed in the position of the decarboxylated methionine moiety in DsSPDS1. In the DsPMT homology model (Figure 2B) the SAM carboxyl group forms two hydrogen bonds with T117 and T144 (numbering of DsPMT) suggesting a third exchange in DsSPDS1, V106T. According to the positioning of SAM in DsSPDS1 like in DsPMT the amino group of SAM will also form a hydrogen bond to D173.

**Figure 3:**
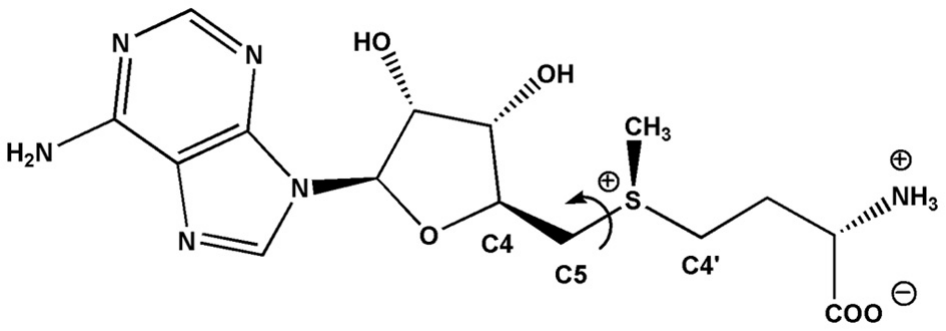
**Conformation of SPDS-bound dcSAM will change in SAM by a rotation around the bond S^+^−C5**.

Thus, the three mutations (Q79T, D103I, V106T) may force a conformational change of methionine in SAM compared to dcSAM in DsSPDS and should lead to an orientation of the methyl group on the sulfur toward the reactive amino group of putrescine. The suggested change of methionine conformation can be measured as torsion angle around the bond between S^+^−C5 (Figure 3). The corresponding angle of dcSAM is −178.9° in DsSPDS1 and switches to −74.78° for SAM.

The altered methionine conformation in SAM will direct the methyl group on the sulfur for transfer onto putrescine instead of the aminopropyl part. L70 in DsSPDS1 corresponds to H108 in DsPMT, which could additionally stabilize the carboxyl group of SAM. This histidine is conserved among PMT sequences. Histidine is similarly also discussed as proton acceptor in several other methyltransferases (Zhang et al., 2000; Zubieta et al., 2001, 2002). Thus L70H was the fourth predicted exchange. In agreement with former suggestions (Korolev et al., 2002; Ikeguchi et al., 2006; Minguet et al., 2008), our models supported the assumption that only a few amino acid exchanges in the methionine binding region are sufficient to change the catalytic activity from an aminopropyl transfer to a methyl transfer.

### *Datura stramonium* spermidine synthase mutants

According to the recommendations derived from homology model comparisons, four amino acids encoded in the DsSPDS1 gene were stepwise exchanged into the equivalent residues of DsPMT (Figures 2A,B). The corresponding proteins were heterologously synthesized in *E. coli*, purified, and tested for their coenzyme acceptance and enzymatic activity (Table 4).

**Table 4:** **PMT activities of DsSPDS1 and DsPMT mutants; DsSPDS1 wild type and DsPMT wild-type activities for comparison**.

**Enzyme**	**Exchanges**	**v_max_[pkat mg^−1^]**	**K_m_ putrescine [μM] K_m_ SAM [μM]**	**k_cat_ [1 s^−1^]**
DsSPDS1 mutant	D103I	24 ± 6.12	n.d.	0.84 x 10^−3^
	D103I,	242 ± 6.32	48 ± 6.68	8.48 x 10^−3^
	Q79T		31 ± 3.42	
	D103I,	4338 ± 170.50	159 ± 27.29	0.15
	Q79T,		27 ± 2.99	
	V106T			
	D103I,	146 ± 17.40	n.d.	5.12 × 10^−3^
	Q79T,			
	V106T,			
	L70H			
DsSPDS1 wild type (Biastoff et al., 2009b)		12,500	33	0.44
			64 (dcSAM)	
DsPMT wild type		51,238 ± 1420	190 ± 27.67	1.98
			13 ± 1.97	
DsPMT mutant	H108L	20,035 ± 661	3130 ± 732.59	0.78
			99 ± 11.68	

None of the mutants showed SPDS activity. n.d., not determined; n = 3–4 ± standard error.

The single exchange of D103I resulted in an inactive SPDS (detection limit 0.96 pkat mg^−1^ protein). A similar finding was observed in *T. maritima* SPDS; after the analogous exchange D101I k_cat_ was ca. 2000-fold reduced, and K_m_ for dcSAM increased (Wu et al., 2007). Here, for the first time, a low methyltransferase activity was detected after D103I exchange with a k_cat_ of less than 0.04% of the DsPMT wild type. The second replacement in DsSPDS1, Q79T, increased the PMT activity to 0.00848 reactions s^−1^, i.e., 0.5 reactions min^−1^. Thus, only two amino acid substitutions in the dcSAM binding site enabled the efficient transfer of the methyl group of SAM to putrescine. Judging from the homology model of DsSPDS1 double mutant D103I and Q79T, the additional exchange of V106T should afford a better binding of SAM due to introduction of a hydrophilic environment and stabilization the methionine carboxyl group in SAM by hydrogen bonds. The triple mutant indeed revealed a catalytic activity of 9 reactions min^−1^ for methyl group transfer. The fourth exchange L70H, however, decreased PMT activity to 0.3 reactions min^−1^ (Table 4). Although the histidine is conserved in all PMT sequences (Teuber et al., 2007), histidine did not appear essential for PMT activity in a mutated DsSPDS1. We exchanged H108L in DsPMT accordingly and found the enzyme to exert PMT activity with ca. 40% maximal turn over velocity (k_cat_) of the wild-type DsPMT. K_m_ for SAM was slightly elevated, but putrescine saturation appeared only at very high concentrations (>6 mM). This indicates that histidine in DsPMT wild type equally is not essential for catalysis, but contributes to putrescine affinity. All DsSPDS1 mutants were analyzed for aminopropyl transfer ability but did not show any catalytic activity with the coenzyme dcSAM (detection limit 0.96 pkat mg^−1^ protein). The increased SAM binding affinity (K_m_) observed with the triple mutant was similar to that of wild type PMT and underlines the importance of these conserved amino acids for the generation of PMT activity in a SPDS protein frame.

The substitution of only one amino acid D103I responsible for coenzyme binding already enabled the transformation of the enzymatic function from SPDS to PMT. Additional exchanges of amino acids in the coenzyme binding region increased the catalytic activity notably. This observation reinforces the hypothesis that PMT evolved from SPDS (Hashimoto et al., 1998a; Minguet et al., 2008). Possibly, a duplicated *spds* gene after a few mutations encoded PMT activity. In the light of the present results, the proposed amino acid exchanges were basically correct; however, protein models currently are not sufficient to predict the effect of each amino acid exchange on the activity and specificity of the resulting enzymes.

### *Arabidopsis thaliana* spermidine synthase mutants

**Figure 4:**
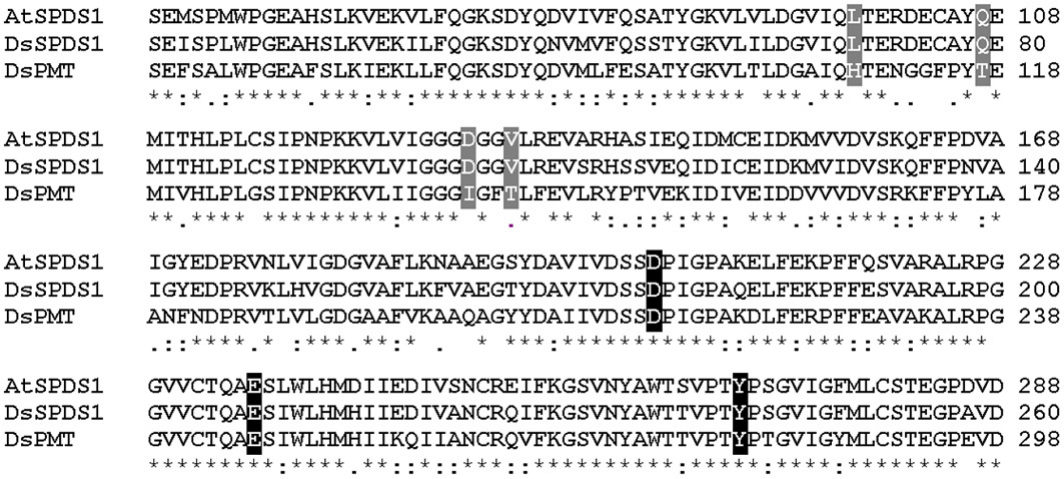
**Sequence alignment of wild type enzymes by Clustal W. (Larkin et al., 2007), sequence identities in amino acid sequences: AtSPDS1 to DsSPDS1 82%, AtSPDS1 to DsPMT 60%, DsSPDS1 to DsPMT 67%**. Changes by site-directed mutagenesis in coenzyme binding site: gray, changes in putrescine binding site: black; Gonnet-Matrix; ^*^single, fully conserved residue; : conservation between groups of strongly similar properties;. conservation between groups of weakly similar properties.

The successful conversion of DsSPDS1 into an active PMT stimulated similar experiments with another SPDS from *A. thaliana* (AtSPDS1) that is 82% amino acid identical to DsSPDS1 (Figure 4). While AtSPDS1 with a single amino acid substitution (D131I) displayed low PMT activity, it was still able to catalyze aminopropyl transferase activity (Figures 2C,D, Table 5). Residual SPDS activity remained after additional exchanges Q107T and V134T (Table 5). In AtSPDS1 the second amino acid exchange chosen as V134T had a more pronounced effect on PMT activity increase than the exchange Q107T. PMT activity after totally three amino acid exchanges increased to ca. 5 reactions min^−1^ comparable to 3-fold mutated DsSPDS1. Obviously, a highly active PMT can be achieved by variable mutation steps in AtSPDS1 wild type, and the sequence of the three amino acid exchanges has some degrees of freedom. Like in DsSPDS1, introduction of histidine, L98H, decreased PMT activity, and SPDS activity was no longer measurable. The enzyme homology models were challenged to show the difference in coenzyme binding capacity between the mutated DsSPDS1 and AtSPDS1 (Figures 2A,C,D). In both proteins, SAM and dcSAM were positioned with similar efficiency, and both coenzymes could be adjusted in a position for the correct group transfer. The homology models were able to indicate the correct amino acid exchanges for activity transit. Prediction of possible side activities will demand further homology modeling, e.g., force field calculations.

**Table 5:** **Enzyme activities of AtSPDS1 wild type and mutants**.

**Enzyme**	**Exchanges**	**SPDS activity**	**PMT activity**		
		**v_max_ [pkat mg^−1^]**	**v_max_ [pkat mg^−1^]**	**K_m_ putrescine [μM] K_m_ SAM [μM]**	**k_cat_ [1 s^−1^]**
AtSPDS1 wild type	–	20,722 ± 351.1	–	220 ± 18.44	0.722
				18 ± 1.23 (dcSAM)	
AtSPDS1 mutant	D131I	29 ± 0.74	119 ± 2.74	56 ± 4.45	4.48 × 10^−3^
				28 ± 0.77	
	D131I,	6 ± 0.33	100 ± 0.75	126 ± 9.22	3.80 × 10^−3^
	Q107T			38 ± 1.43	
	D131I,	14 ± 1.28	250 ± 2.18	85 ± 5.24	9.51 × 10^−3^
	V134T			65 ± 2.54	
	D131I,	4 ± 0.58	2155 ± 17.55	32 ± 1.88	0.081
	Q107T,			12 ± 0.60	
	V134T				
	D131I,	<d.l.	144 ± 1.61	100 ± 7.67	5.47 × 10^−3^
	Q107T,			50 ± 2.65	
	V134T,				
	L98H				

<d.l., below detection limit; n.d., not determined; n = 3–4 ± standard error.

### Binding regions of putrescine and SAM in mutants of AtSPDS1

All exchanges of amino acids in DsSPDS1 and AtSPDS1 so far were located in the coenzyme binding region. Returning to the hypothesis of a different binding region of putrescine for the development of PMT activity (Biastoff et al., 2009b), mutations were designed in the putrescine binding cavity now equally assumed for SPDS and PMT (Figure 2). AtSPDS1 wild type and the AtSPDS1 double mutant with D131I and Q107T (AtSPDS1-D131I-Q107T) were chosen because protein synthesis in *E. coli* was experienced as more efficient than for DsSPDS1 and its mutants, and sequence identity is high (Figure 4). In homology models and alignments, D204 (AtSPDS1 numbering) is totally conserved in SPDSs and PMTs. In the human SPDS (PDB: 2O06), a hydrogen bond of an aspartate equivalent to D204 to the non-reacting amino group of putrescine was observed (Wu et al., 2007). Our homology models indicated a further salt bridge between E236 and the non-reacting amino group of putrescine. Y270 may stabilize the reacting amino group of putrescine by a hydrogen bond, while the hydrophobic phenyl ring possibly interacts with the butyl chain of putrescine. If putrescine is located in the identical binding region in SPDSs and PMTs, the exchange of stabilizing amino acids should lead to activity reduction. Thus the residues D204, Y270, and E236 were replaced by alanine. D204 was equally replaced by lysine, inferring that if D204 resides at the end of the putrescine binding cleft, a lysine at that position will protrude with the amino end into the putrescine binding cavity and, by inhibiting putrescine binding completely, abolish enzyme activity.

The mutated enzymes showed (Table 6) that exchange of D204 in AtSPDS1 wild type to alanine caused a 20-fold reduction of aminopropyl transferase activity. After replacement of D204K in wild type, SPDS activity was totally lost. After identical exchanges in the AtSPDS1 double mutant D131I-Q107T, PMT activity was not detectable anymore. Replacement of Y270A caused a severe reduction of PMT activity. The exchange of E236A reduced the PMT activity 5-fold. The experimental results complied with the expectations from modeling and concordantly supported the concept of putrescine residing in the same binding location after mutation from SPDS to PMT activity.

**Table 6:** **Enzyme activities in AtSPDS1, wild type and double mutant, resulting from mutated amino acids that were considered as important for binding region of putrescine**.

	**Exchanges**	**SPDS activity k_cat_ [1 s^−1^]**	**PMT activity k_cat_ [1 s^−1^]**
AtSPDS1 wild type	D204A	0.034	Not measured
	D204K	<d.l.	
AtSPDS1 mutant	D204A	Not measured	<d.l.
D131I, Q107T	D204K		<d.l.
	Y270A		1.43 × 10^−4^
	E236A		8.21 × 10^−4^

<d.l., below detection limit; n = 3–4.

## Discussion

The evolution of plant metabolic diversity is an enigma, for which many theories exist. The transition from SPDS to PMT is discussed as a typical example for the change of function (neofunctionalization; Ohno, 1970) after gene duplication and adapting mutations (Minguet et al., 2008; Biastoff et al., 2009a; Ober, 2010). Time windows for adaptations of duplicated genes, however, are small, as two genes with identical functions are unlikely to be stably maintained in a genome and most mutations will lead to rapid non-functionalization. For the diversification of enzyme functions, the concept of subfunctionalization was set against Ohno's model implying that many enzymes catalyze various reactions (Khersonsky and Tawfik, 2010). After gene duplication, both copies diverge by optimization for one function. In general, the genetic fixation of duplicated genes is dependent on the ease, by which gene copies attain differentiated functions (Innan and Kondrashov, 2010). The identification of possible PMT evolutionary steps serves as an experimental proof for both theoretical models of evolutionary diversification. In our experiments, DsSPDS1 changed its function after one amino acid exchange, while in AtSPDS1 a double-functional enzyme postulated for the concept of subfunctionalization was produced. We therefore comply with the recently formulated view that in nature sub- and neofunctionalization are difficult to distinguish and are not mutually exclusive (Flagel and Wendel, 2009; Ober, 2010); they may occur simultaneously in specialization.

The initial PMT activity that we achieved by mutating SPDS was low in both SPDS enzymes. For providing a new metabolic trait such low activity will suffice presuming that SPDS is ubiquitous in plant cells and highly inducible in stress conditions (Alcazar et al., 2011). Temporary high enzyme levels may effectuate a low side activity and produce an additional metabolite. One or two additional amino acid exchanges can provide an efficient PMT activity.

How can a new metabolite, *N*-methylputrescine, for which no advantage in selection is known, provide the foundation of a new metabolic pathway to final alkaloids with repellent or protective functions? For the evolution of chemical diversity, the participation of broad substrate enzymes was recently postulated (Weng et al., 2012). Assuming that unspecific diamine oxidases present in many plant tissues will oxidize *N*-methylputrescine, the resulting *N*-methylpyrrolinium cation is a reactive metabolite ready for various condensations. Alkaloids containing a *N*-methylpyrrolinium moiety are widespread in plants, not only in tropane and nicotine alkaloids. Tropane alkaloids already are found sporadically in many families of dicot plants distinct from Solanaceae and Convolvulaceae, e.g., in Proteaceae, Brassicaceae, and Rhizophoraceae (Lounasmaa and Tamminen, 1993). Examples for further alkaloids containing the *N*-methylpyrroline moiety are numerous in plants, e.g., hygrine and shihunine in *Dendrobium* species (Orchidaceae, a monocot) (Luening and Leander, 1965; Leete and Bodem, 1976) or mesembrine and mesembrinine in *Sceletium* species (Aizoaceae, Caryophyllales) (Popelak et al., 1960). The frequent occurrence of putative *N*-methylputrescine derivatives and the few mutations that are needed to generate PMT activity in SPDS proteins leads us to speculations. Possibly, in numerous proteins annotated as SPDS, PMT activity may reside exclusively or as side activity. Sequence comparisons alone did not reveal the activity specificities; a combination with rigorous *in vitro* examination with high sensitivity for enzyme products was successful.

**Figure 5:**
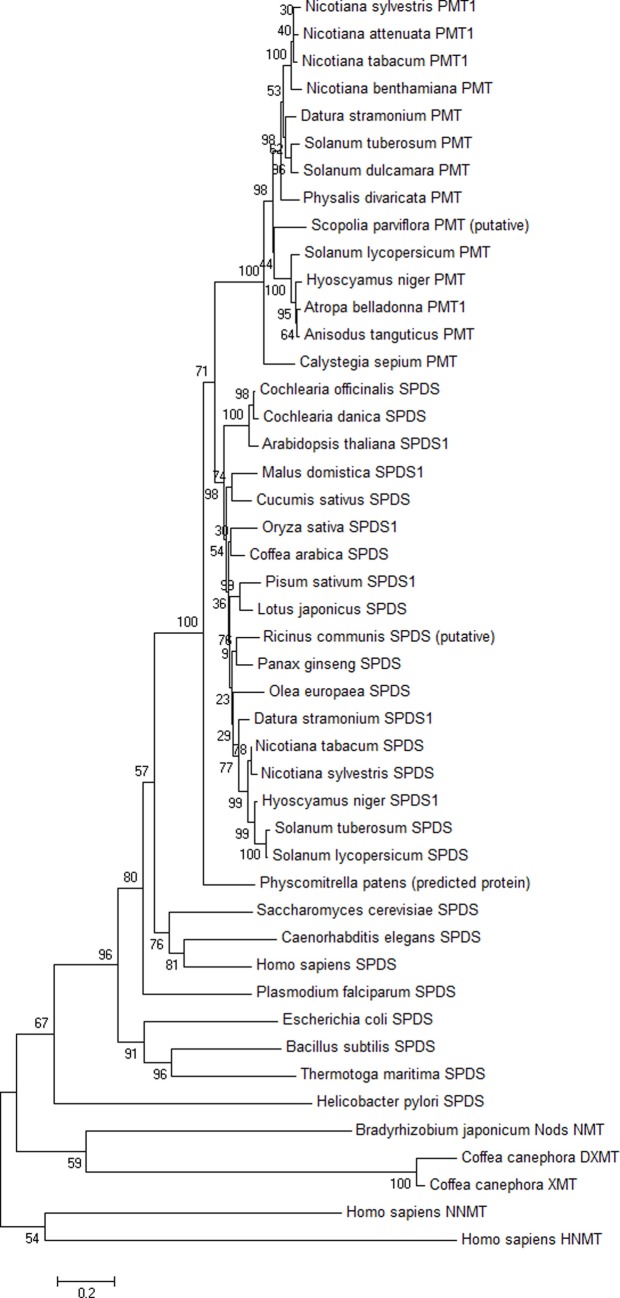
**Phylogenetic tree of PMT, SPDS, and N-methyltransferase (NMT) proteins**. The unrooted tree was computed with MEGA5 (Tamura et al., 2011) using the Neighbor-Joining method. Accession numbers of the sequences are given in Table 3. Bootstrap values (1000 replicates) are shown next to the branch points. Bar length indicates 0.2 exchanges per residue.

Locating PMT amino acid sequences within a phylogenetic tree of SPDS sequences (Figure 5) reveals a branch of PMT that diverges at the base of all plant SPDS. *N*-Methyltransferases were integrated in the tree for comparison; they differ strongly from PMT and SPDS. PMT sequences for the tree were obtained from all plant genera known to date to contain PMT. All PMTs originate from Solanaceae except one from *Calystegia sepium*, a Convolvulaceae that is systematically close to Solanaceae in the order of Solanales. It could be assumed therefore that the PMT branch diverges out of a branch of Solanales SPDSs. The branch point was found, however, between PMT and Flowering plant (Angiosperm) SPDS. A moss (*Physcomitrella patens*) protein resembling SPDS is located directly below the branch point and this branch is well supported. The resolving power of the NJ tree within higher plant sequences is not high enough to locate the PMT origin definitely at the higher plant SPDS basis. Bootstrap values are low for some branch points within SPDS, and the branch between Angiosperm SPDS and PMT is supported by 71. We constructed additional trees (not shown), e.g., by omitting plant *N*-methyltransferases, by omitting PMTs, by adding further plant SPDSs, and the branch point between PMTs and all Angiosperm SPDSs remained the same, with similar bootstrap values ranging between 53 and 100. We conclude that the PMT known to date are monophyletic and developed within higher plants. It remains to be elucidated whether PMT occurs in further unrelated families of higher plants and whether those so far undetected PMTs are similar to the current PMT sequences or whether they form new groups.

### Conflict of interest statement

The authors declare that the research was conducted in the absence of any commercial or financial relationships that could be construed as a potential conflict of interest.
